# Mapping the landscape of mental health research through Google Trends: Bibliometric and thematic insights

**DOI:** 10.1002/pcn5.70101

**Published:** 2025-04-27

**Authors:** Abhishek Ghosh, Blessy B. George, Pragyapti Malav, Shinjini Choudhury, Sonu Goel

**Affiliations:** ^1^ Department of Psychiatry Postgraduate Institute of Medical Education and Research (PGIMER) Chandigarh India; ^2^ Department of Psychiatry All India Institute of Medical Sciences Patna Bihar India; ^3^ Department of Community Medicine & School of Public Health Postgraduate Institute of Medical Education and Research (PGIMER) Chandigarh India; ^4^ Faculty of Education & Health Sciences University of Limerick Limerick Ireland; ^5^ Faculty of Human and Health Sciences Swansea University Wales UK

**Keywords:** bibliometric analysis, content analysis, Google Trends, mental health

## Abstract

**Aim:**

The rise of Internet usage has introduced innovative methods for public health research, particularly using Google Trends to understand mental health issues. This study aims to conduct a comprehensive bibliometric and content analysis of research utilizing Google Trends for mental health.

**Methods:**

We conducted a literature search on Scopus focusing on peer‐reviewed articles from January 2010 to May 2024. Bibliometric analysis included descriptive statistics, bibliographic coupling, keyword co‐occurrence, and coauthorship networks. Qualitative content analysis identified themes in the study objectives.

**Results:**

The bibliometric analysis revealed an increase in publications post‐pandemic. The bibliographic coupling analysis identified the *Journal of Medical Internet Research* as central, with significant connections to journals like *JMIR Public Health and Surveillance* and *BMC Public Health*. Keyword co‐occurrence highlighted themes such as “mental health,” “COVID‐19,” “anxiety,” and “social media.” Author co‐citation and coauthorship analyses showed strong closely linked collaborations, with a few central authors leading the research. Coauthor country analysis revealed limited international collaborations, particularly involving the United States and the United Kingdom. Content analysis identified six major themes: economic and social impacts, mental health during public health emergencies, online behavior, specific conditions and treatments, public health policies, and psychological and social impacts.

**Conclusion:**

This study underscores the importance of Google Trends in mental health research, revealing key trends and thematic focuses. The findings contribute to understanding the current research landscape and offer a foundation for future studies leveraging digital tools for mental health insights.

## INTRODUCTION

The rapid proliferation of Internet usage ushered in a new era for public health research, that is, digital epidemiology, which refers to the use of digital data sources (e.g., search engine queries, social media interactions, mobile health data) for monitoring, predicting, and analyzing health trends. Among the various tools used in digital epidemiology, Google Trends has emerged as a pivotal resource for understanding public interest and behavior concerning mental health issues. This tool, which tracks the frequency of search terms over time, offers a unique lens through which researchers can analyze trends, predict outbreaks, and identify public concerns related to mental health.^1^, ^2^ Despite its potential, Google‐Trends‐based research faces methodological challenges, including demographic biases, differences in search behaviors across populations, and the inability to validate searches as proxies for actual disease prevalence.^1^ Nonetheless, the integration of Google Trends data into mental health research is an innovative approach that bridges the gap between traditional epidemiological methods and contemporary digital behavior analysis.

The application of Google Trends in mental health research has gained considerable traction over the past decade. Researchers have leveraged this tool to investigate a myriad of mental health conditions, ranging from anxiety and depression to substance use disorders and suicide.^3^, ^4^ The rationale behind utilizing Google Trends is rooted in the hypothesis that the frequency and pattern of specific search queries can reflect real‐world behaviors and concerns.^5^ For instance, an uptick in searches related to “depression symptoms” or “anxiety treatment” may indicate a corresponding increase in the prevalence or awareness of these conditions within the population.

Bibliometric analysis involves the quantitative evaluation of publications, providing insights into the volume of research, prominent themes, influential authors, and key journals in the field.^6^ Content analysis, on the other hand, would delve into the qualitative aspects of the study objectives, revealing the underlying motivations, hypotheses, and research questions that drive this burgeoning area of research.^7^ The bibliometric analysis in this study focuses on quantifying the growth of literature utilizing Google Trends for mental health research. This involves assessing the number of publications over time, identifying leading authors and institutions, and mapping the most influential journals and conferences.^8^ Such an analysis is crucial for understanding the development of the field, highlighting areas of significant research activity, and identifying potential gaps that warrant further investigation. Bibliometric analysis has been successfully applied in other health domains, including chronic diseases like sarcopenia.^9^ Azizan^9^ demonstrated that bibliometric network analysis could effectively map research trends, collaborations, and knowledge structures in sarcopenia research, identifying key themes and influential contributors. Drawing a parallel, our study aims to map the intellectual landscape of Google‐Trends‐based mental health research, identifying emerging themes, influential authors, and collaborative networks.

Content analysis may complement the bibliometric approach by examining the specific objectives of the studies included in the review. Qualitative analysis categorizing the research objectives into several themes could help identify key research areas. Additionally, the analysis can explore specific mental health conditions and treatment strategies, public health policies, and the broader psychological and social impacts of mental health issues. With this background, this study aims to conduct a comprehensive bibliometric and content analysis of the research objectives in studies that have employed Google Trends to investigate mental health and related concerns.

One of the significant motivations for this study is the recognition that traditional epidemiological methods may not fully capture the dynamic and rapidly changing landscape of mental health concerns. Google Trends offers a real‐time, data‐driven approach to understanding public interest and behavior, providing valuable insights that can inform public health interventions, policy decisions, and future research directions.^10^ By synthesizing the findings from bibliometric and content analyses, this study aims to provide a holistic view of the current state of Google‐Trends‐based mental health research, identifying key trends, common research themes, and potential areas for further exploration. Further, the integration of digital tools like Google Trends into mental health research represents a paradigm shift, reflecting the broader trend toward digital epidemiology and big‐data analytics in public health. Through this comprehensive analysis, we aim to contribute to the growing body of knowledge on digital health research and provide a foundation for future studies that seek to leverage digital data for mental health insights.

## METHODS

### Study design

This study employs a mixed‐methods approach, integrating both bibliometric and content analysis to comprehensively examine the objectives of Google‐Trends‐based mental health research. The mixed‐methods design facilitates a holistic understanding of the research landscape by combining quantitative evaluation with qualitative insights.

### Data collection

We conducted a literature search on the Scopus database on June 26, 2024. Scopus provides a broad coverage of peer‐reviewed publications relevant to Google Trends and mental health. Scopus was chosen due to its extensive indexing of interdisciplinary journals, structured metadata, and citation analysis tools, making it well‐suited for bibliometric studies. While databases such as Web of Science and PubMed also index relevant literature, Scopus was selected to ensure consistency, minimize duplication, and facilitate network mapping in bibliometric analysis. The search terms included “google trends,” “mental health,” “depression,” “anxiety,” “mental disorder,” “psychological well‐being,” “mental illness,” and “psychiatric disorder.” An initial list of terms was compiled based on established literature and refined through expert consensus. Two of the coauthors, who are mental health professionals, reviewed and validated the keywords to ensure their comprehensiveness. In line with best practices in bibliometric research,^11^ alternative terms, such as “mental wellness,” “psychological well‐being,” and “emotional health,” were incorporated to enhance inclusivity. While broadening the keyword list further may have increased recall, we carefully balanced specificity and thematic relevance to maintain methodological rigor.

The search was limited to peer‐reviewed articles published between January 2010 and May 2024.

### Selection criteria

To ensure the relevance and quality of the included studies, the following inclusion criteria were applied: studies that utilized Google Trends data to investigate mental health issues, peer‐reviewed journal articles, research letters, and review papers, studies published in English, and studies providing clear objectives related to the use of Google Trends in mental health research.

### Exclusion criteria

Studies not related to mental health, opinion pieces, editorials, and commentaries were excluded from the analysis.

### Study selection process

Since all records were retrieved from a single database (Scopus), duplicate removal was not required. Title and abstract screening, followed by full‐text assessment, was conducted independently by two reviewers (P. M., B. B. G.) to determine eligibility. Discrepancies were resolved through discussion or consultation with a third researcher (A. G.) when necessary. Studies were included if they focused on Google Trends in mental health research and excluded if they were non‐empirical or unrelated to mental health.

### Bibliometric analysis

Descriptive statistics: We gathered the number of publications per year and the types of publications. Bibliographic coupling: Bibliographic coupling is when two documents cite one or more common references, indicating subject matter similarity. Sources with at least two common citations were included:

Keyword co‐occurrence analysis: This is the identification of common keywords and their relationships to uncover major research themes and trends. Keywords used in a minimum of five sources were included.

Coauthorship and collaboration networks: Under this, we generated av isual mapping of research collaborations among authors and institutions. Citation analysis examined the impact of authors or publications, including those cited at least twice. Co‐citation analysis identified frequently cited pairs of documents or authors. Coauthorship analysis examined collaborative relationships among authors, including those who published at least two articles. Coauthor country analysis revealed international collaborations, including countries with at least three publications. Coauthor organization analysis examined institutional collaborations, including organizations with at least two studies.

Funding sources mapping: We categorized into government agencies (e.g., National Institute for Health Research [NIHR]), universities and research institutions (e.g., Cardiff University), foundations and trusts (e.g., Wellcome Trust), and private companies and organizations (e.g., Google).

We generated “connected” maps for all metrics using the “association strength” method to generate network maps and clusters.

### Content analysis

A qualitative content analysis was performed to identify and categorize the study objectives mentioned in the abstracts. This analysis aimed to uncover the underlying motivations, research questions, and hypotheses using Google Trends in mental health research. The process included the following.

#### Open coding to identify key themes and concepts

Initial coding was conducted using ATLAS.ti software. B. B. G. and P. M., both with backgrounds in psychiatric social work and public health, independently coded the study objectives. They discussed and reached a consensus on the codes, removing redundancies, merging similar codes (e.g., “economic downturn” and “financial problem”), and shortening lengthy code names. Codes that could not be agreed upon were discussed with A. G. to finalize the code list.

#### Axial coding to group similar codes into broader categories

This step was initially performed using ATLAS.ti software and involved grouping similar codes into overarching themes. A. G. reviewed and regrouped some codes to better fit the themes.

#### Selective coding to refine the themes

This step ensured the themes accurately represented the study objectives. Themes were framed to capture mental health, disorders, well‐being, psychosocial concerns, population interest, and public health and policy.

The iterative reading and re‐reading of the extracted objectives ensured comprehensive theme identification and accurate representation of the study objectives.

## RESULTS

We identified 159 articles and excluded eight (five conference papers and one book chapter, one editorial, and one conference review). The first article using Google Trends for mental health research was published in 2014 and most articles (*n* = 23) were published in 2023. There has been a gradual increase in the number of published articles in the last 10 years; 2020 witnessed the sharpest peak (please see Figure S1).

### Results of the bibliometric analysis

#### Bibliographic coupling

We generated a network visualization of bibliographic coupling among journals. Each node represents a journal, with the node size indicating the volume of publications. Larger nodes correspond to journals with more publications. The edges connecting the nodes represent the strength of bibliographic coupling, with thicker lines indicating stronger connections, meaning the journals share more common citations. The color clusters in the figure represent groups of journals closely related by citation patterns, indicating thematic similarities and research communities.

**Figure 1:**
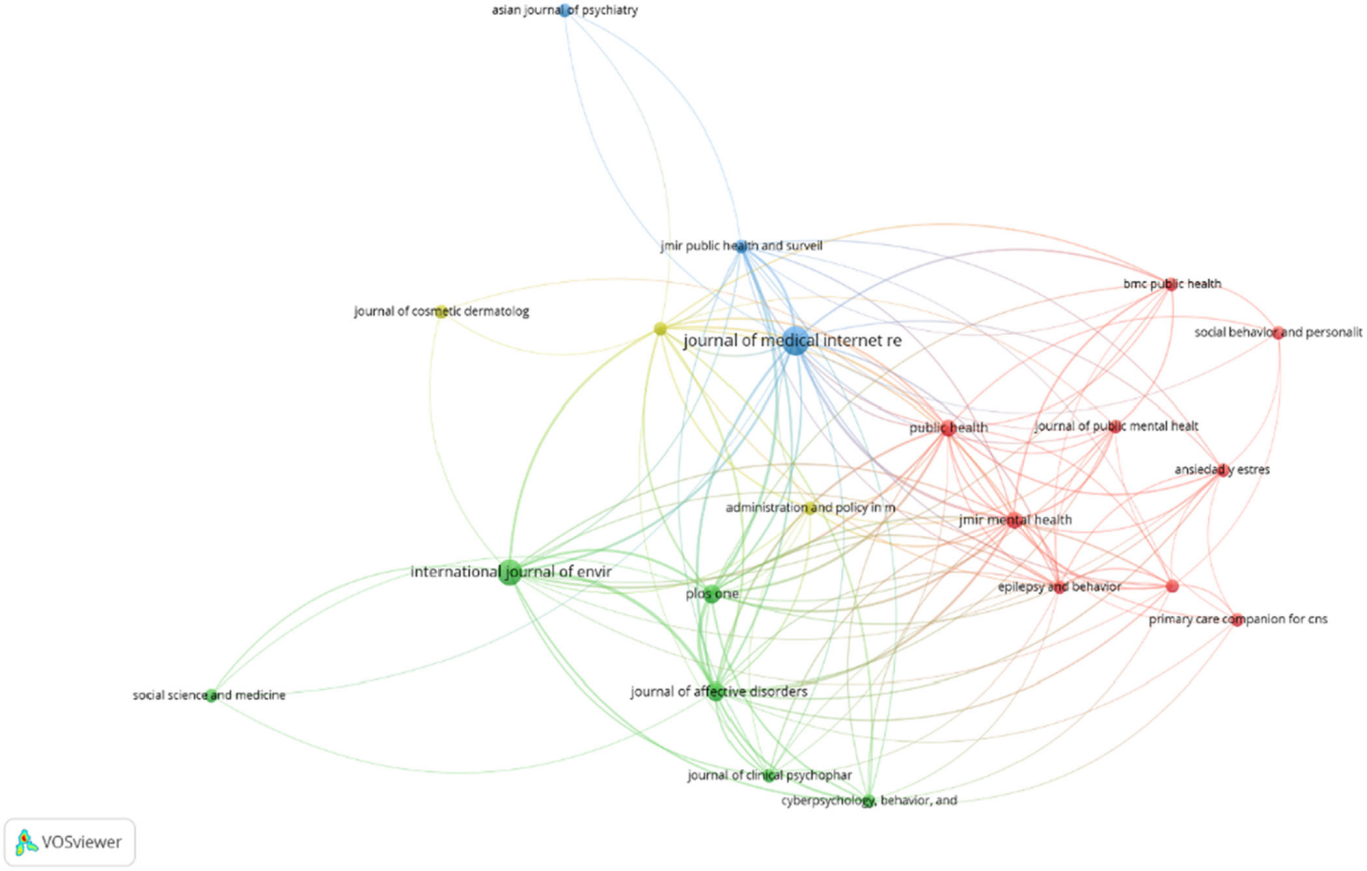
Bibliographic source analysis.

The blue cluster is dominated by the *Journal of Medical Internet Research* (JMIR), including journals such as *JMIR Public Health and Surveillance*, and *JMIR Mental Health*, focusing on digital health, Internet research, and public health surveillance. The red cluster includes *BMC Public Health*, *Social Behavior and Personality*, and *Journal of Public Mental Health*, highlighting intersections of mental health and public health. The green cluster, centered around the *International Journal of Environmental Research and Public Health*, includes *Social Science & Medicine* and *Journal of Affective Disorders*, suggesting a focus on environmental factors and broader social science research. Other notable nodes, such as the *Asian Journal of Psychiatry* and the *Journal of Cosmetic Dermatology*, indicate specialized research areas with fewer connections. Therefore, the journals focusing on digital medicine, health, and healthcare and public health are more interested in Google‐Trends‐based mental health research (Figure 1).

Figure 1 illustrates a network visualization of bibliographic coupling among journals. Each node represents a journal, with node size indicating publication volume. Edges indicate the strength of coupling, with thicker lines representing more common citations. Color clusters denote thematic similarities: blue for digital health, red for public health and social behavior, green for environmental factors and social sciences, and isolated nodes for specialized research areas. This visualizes how journals are interlinked through shared citations, highlighting major research communities.

#### Keyword concurrence

Each node in the network represents a keyword, with the node size indicating its frequency within the dataset. Larger nodes correspond to more frequently appearing keywords. The edges connecting the nodes represent co‐occurrence relationships, with thicker lines indicating stronger connections, meaning the keywords frequently appear together in the same studies.

The color clusters represent groups of keywords closely related based on co‐occurrence patterns, indicating thematic similarities. The red cluster includes central keywords, such as “mental health,” “humans,” “human,” “article,” “search engine,” and “coronavirus disease 2019,” highlighting a focus on mental health issues during the COVID‐19 pandemic. This cluster also includes “depression,” “anxiety,” “suicide,” and “social media,” reflecting the psychological impacts and social media's role in mental health research during the pandemic.

The green cluster features keywords such as “pandemic,” “public health,” “psychology,” “epidemic,” and “lockdown,” indicating research on the broader public health implications of the pandemic and its psychological effects. The blue cluster contains keywords like “infodemiology,” “vaccination,” “medical information,” and “global health,” emphasizing research on health information dissemination and vaccination. The yellow cluster includes keywords such as “suicidal ideation,” “unemployment,” “mortality rate,” and “trend analysis,” focusing on specific mental health conditions, socioeconomic factors, and epidemiological trends.

Notable keywords, such as “India,” “United States,” “Canada,” “United Kingdom,” and “Australia” indicate frequently studied geographical locations. Keywords like “autism,” “psychiatry,” “schizophrenia,” and “anxiety disorder” suggest specific mental health conditions focal in the literature.

**Figure 2:**
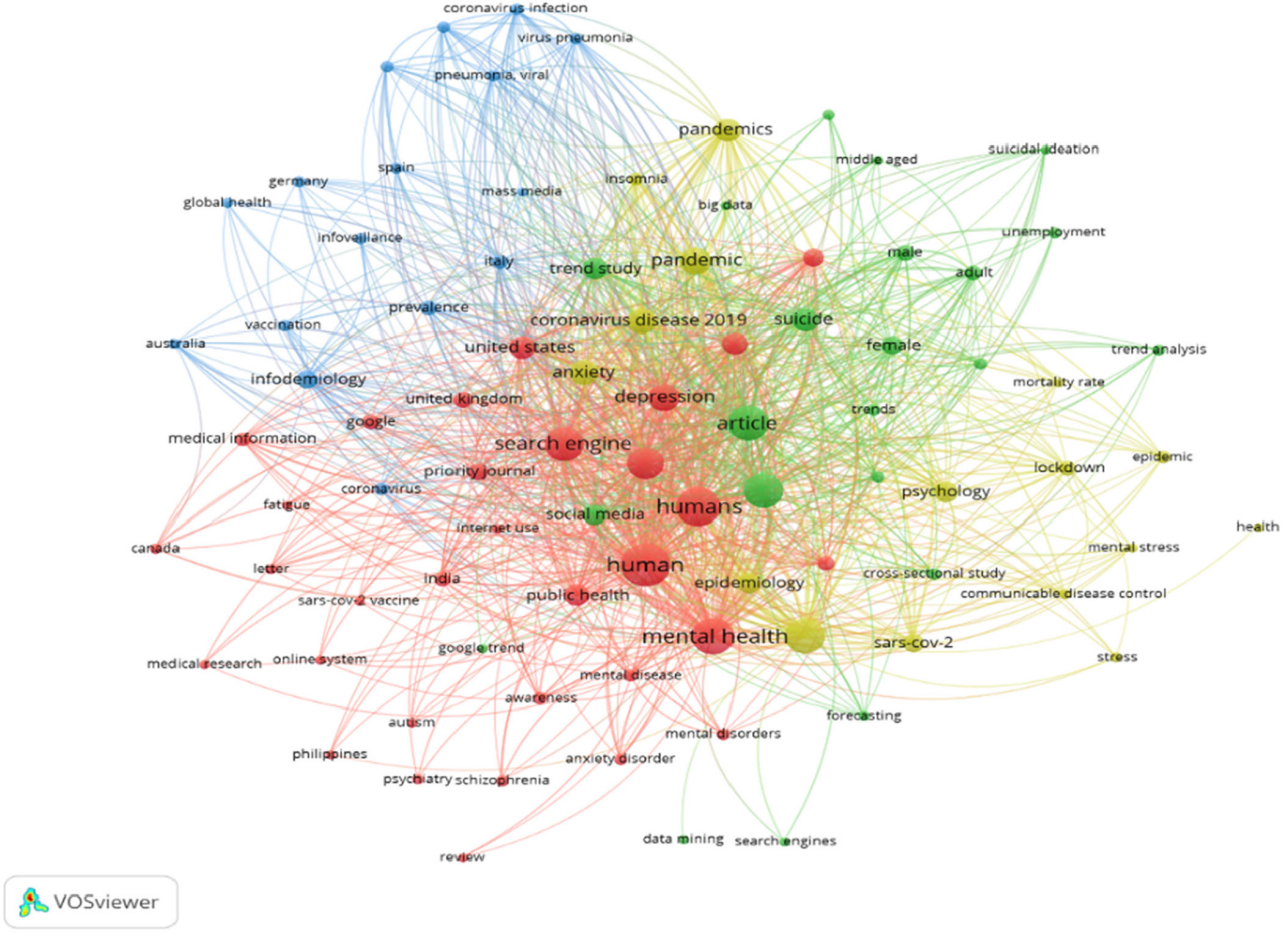
Keyword concurrence.

Therefore, the red, green, and blue clusters represent “broad areas of research and mental health conditions,” “public health response and impact,” and “countries of origin,” respectively (Figure 2).

Figure 2 is a network visualization of keyword co‐occurrence in mental health research using Google Trends and shows clusters of related themes. Red highlights COVID‐19's mental health impact, green focuses on public health implications, blue on health information dissemination, and yellow on specific conditions and socioeconomic factors.

#### Author co‐citation analysis

Each node represents an author, with the node size indicating citation frequency. Edges represent co‐citation relationships, with thicker lines indicating stronger connections, meaning the authors are frequently cited together. The network has two main clusters.

**Figure 3:**
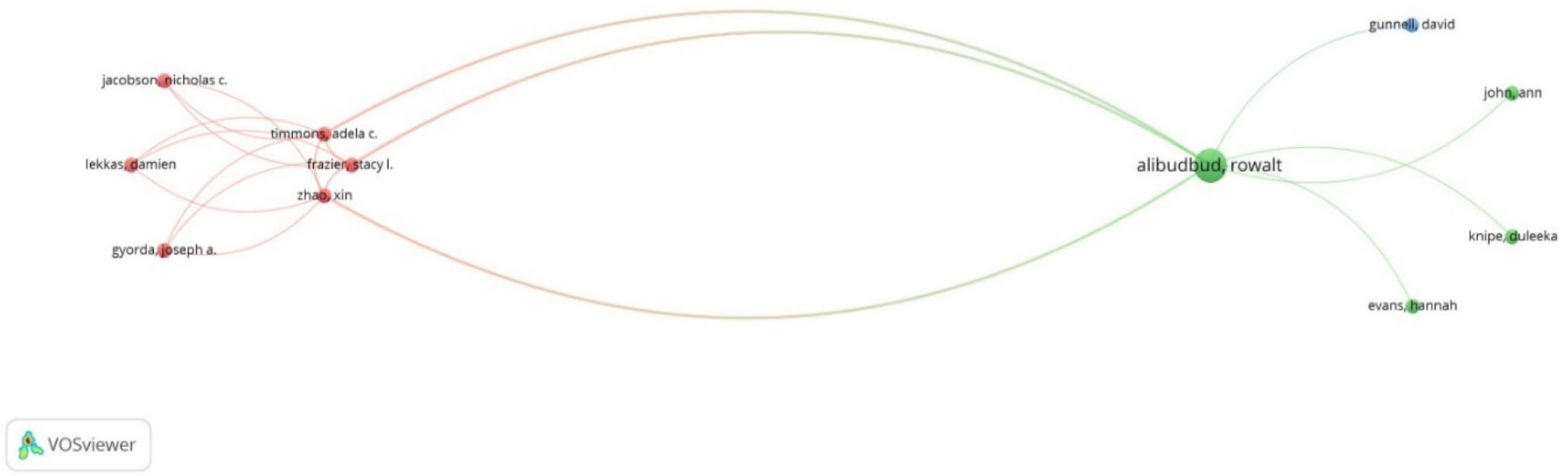
Author co‐citation analysis.

The red cluster includes authors like Nicholas C. Jacobson, Adela C. Timmons, and Stacy L. Frazier, suggesting a collaborative community focused on similar mental health themes. The green cluster centers around Rowalt Alibudbud and includes David Gunnell, Ann John, and Duleeka Knipe, indicating another prominent research community with strong internal co‐citation links. Rowalt Alibudbud's central position suggests significant influence and frequent citation (Figure 3).

This co‐citation analysis map shows two main clusters of frequently cited authors in mental health research. The red cluster includes Nicholas C. Jacobson and colleagues, while the green cluster centers around Rowalt Alibudbud. The map illustrates strong internal co‐citation links within each cluster, highlighting collaborative research communities.

#### Author coauthor analysis

Each node represents an author, with the node size indicating the frequency of their publications within the dataset. The edges connecting the nodes represent coauthorship relationships, with thicker lines indicating stronger collaborations between authors. The visualization shows a small, closely knit network of authors, including Sara Barbalito, Teresa Sánchez‐Gutiérrez, Juan Antonio Becerra‐García, and Ana Calvo. These authors are all interconnected, suggesting a collaborative research group focused on similar themes within mental health research. The central position of Sara Barbalito, with connections to all other authors, indicates her significant role in this collaborative network. The tightly clustered nature of this figure reflects the collaborative efforts of a specific research team.

Therefore, only a single closely knit research community has emerged in our analysis (please see Figure S2).

#### Author co‐citation analysis

Each node represents an author, with size indicating citation frequency. Edges represent co‐citation relationships, with thicker lines indicating stronger connections. The visualization reveals two main clusters.

The red cluster includes Nicholas C. Jacobson, Adela C. Timmons, and Stacy L. Frazier, suggesting frequent co‐citation and a related research focus. The green cluster centers around Rowalt Alibudbud, with strong co‐citation links to David Gunnell, Ann John, Duleeka Knipe, and Hannah Evans. Rowalt Alibudbud's central position, indicated by the largest node and numerous co‐citation connections, highlights his significant influence in the network (please see Figure S3).

#### Coauthor country analysis

Each node represents a country, with size indicating publication frequency. Edges represent coauthorship relationships, with thicker lines indicating stronger collaborations. The visualization reveals several clusters.

The United States, the largest blue node, is the most central, showing extensive collaborations with Taiwan, Peru, and others. The United Kingdom, in red, collaborates significantly with France, Germany, Spain, Mexico, and India. Italy, in yellow, has strong coauthorship links with the Netherlands. The green cluster includes Australia, Poland, and Canada, highlighting their collaborative ties.

**Figure 4:**
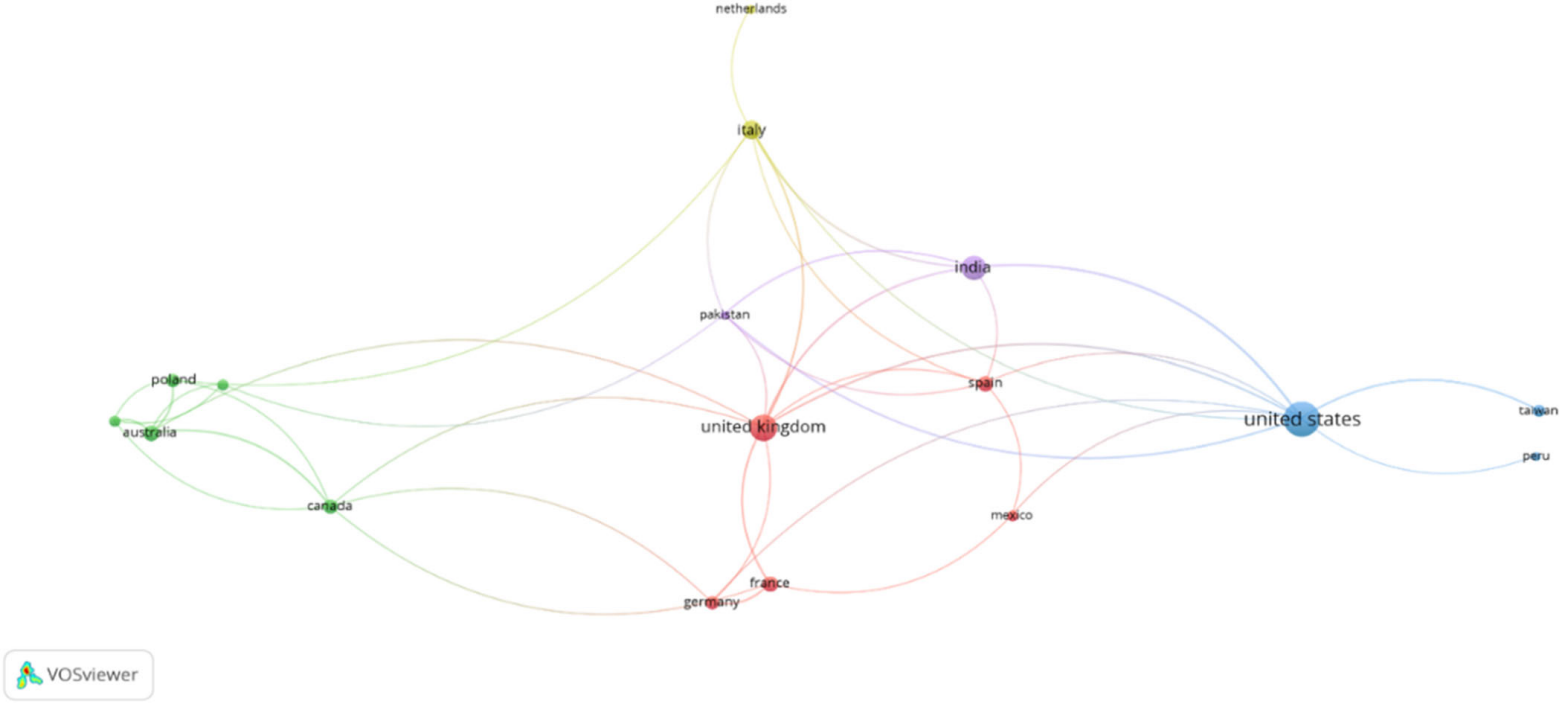
Coauthor country analysis.

Therefore, most studies are from the global north, specifically from the North American and European regions (Figure 4).

#### Coauthor country analysis network visualization

Each node represents a country, sized by publication frequency. Edges show coauthorship relationships, with thickness indicating collaboration strength. The United States (blue) is central, with strong links to various countries. The United Kingdom (red), Italy (yellow), and clusters like Australia–Poland–Canada (green) highlight significant international collaborations.

#### Coauthor organization analysis

Each node represents an organization, with the size of the node indicating the frequency of publications involving authors from that organization. The edges connecting the nodes represent coauthorship relationships, with thicker lines indicating stronger collaborations between organizations.

In this visualization, three organizations are highlighted: the Center for Technology and Behavior, the Department of Psychiatry at Geis, and Quantitative Biomedical Sciences. The Center for Technology and Behavior and the Department of Psychiatry at Geis are closely connected, indicating a strong collaborative relationship. Additionally, there is a notable connection between the Department of Psychiatry at Geis and Quantitative Biomedical Sciences, suggesting another significant partnership.

Therefore, there are only a handful of institutions collaborating in the Google‐Trends‐based mental health research (please see Figure S4).

#### Funding support

Eighty‐seven of the 151 studies were funded research. The National Institute of Health Research, National Institute of Drug Abuse (NIDA), National Institute of Health (NIH), and Wellcome Trust each funded three studies. The European Regional Development Fund and the National Natural Science Foundation of China funded two studies each. The rest of the funding agencies funded single research. Per the categories, the government agencies funded most research (*n* = 45), followed by universities and research institutions (*n* = 23), foundations and trusts (*n* = 12), and private organizations (*n* = 7) (please see Table 1 for further details).

**Table 1:** Content analysis: Codes and themes.

Excerpts	Codes	Themes
“This study assesses the unemployment and mental health correlation and, specifically, whether depression and anxiety increased due to the rise in unemployment during the 2009 debt crisis in France.”	Unemployment, Mental health, Anxiety, Depression	Economic and Social Impact on Mental Health
“This study identifies publicized incidents of racial violence and quantifies national interest based on Google searches; incidents include police killings of Black individuals, decisions not to indict or convict the officer involved, and hate crime murders.”	Discrimination	
“We address in this study the issue of how to proxy sleep and explore sleep's significance for financial markets. We employ daily Google search activity on sleepiness terms (e.g. sleep deprivation) to develop an index and find that a one‐day lagged sleepiness index is related negatively to US stock market returns.”	Sleepiness, Financial,	
“To investigate whether heightened economic uncertainty increases concerns about mental health in the U.S. To achieve this, the study constructs a composite Mental Health Concerns index based on the intensity of Google search queries related to mental disorders and distress.”	Economic impact, Mental health	
“The objective of the study is to examine the commonalities and differences in public mood changes during the COVID‐19 pandemic under different cultural contexts, using China and the United States as representative countries of tight and loose cultures, respectively.”	Mood changes, COVID‐19, Cultural variation	Mental Health During Public Health Emergencies
“The goal of this retrospective, longitudinal time‐series study is to understand the relationship between temporal trends of interest for the search term ‘suicide’ and those of COVID‐19‐related terms, such as ‘social distancing’, ‘school closure’, and ‘lockdown’.”	Suicide, COVID‐19, Social distancing, School closure, Lockdown	
“The objective of the study is to assess the public interest in religion and spirituality (R/S) during the COVID‐19 pandemic to understand the pandemic's impact on societal beliefs. By querying selected keywords related to R/S, COVID‐19, and non‐communicable diseases from Google Trends over a five‐year interval, the study aims to analyze the relationship between the search volumes of these terms. The study performs further statistical analysis to compare cumulative normalized search volumes year by year. The goal is to explore how the search for COVID‐19 and R/S keywords during 2020 reflects a coping mechanism, provides evidence against the common view of the problem of evil, and aligns with previous religious economy studies.”	Religion, Spirituality, COVID‐19,	
“To explore the correlation between COVID‐19 vaccination choice and public mental health during the period from August/2020 to December/2021.”	COVID‐19, Vaccination, Mental health	
“By using such databases, we believe that the question whether changes in cannabis use are associated with changes in schizophrenia rates can be answered, at least partly. Therefore, we tested these tools by evaluating trends in cannabis use and both cases and prevalence of schizophrenia in the United Kingdom, one of the countries where the incident rates for psychotic disorder have been suggested to be particularly high.”	Substance use, Cannabis use, Schizophrenia, Mental health	Specific Mental Health Conditions and Treatment
“Understanding the drivers of the prevalence and spread of biophobias in modern societies is, therefore, a growing concern.”	Biophobias	
“The aims of this study were to expand upon this scarce knowledge by investigating the relationship between web search query volumes and prescription volumes of antidepressants and antipsychotics in the United Kingdom and the Netherlands and to gain insight in topics of concern, such as withdrawal symptoms and discontinuation.”	Antidepressants, Antipsychotics, Prescription volume	
“We have two study objectives: to use Google Trends to identify the annual time points from 2015 to 2019 with the highest Google search traffic in Spain for the terms ‘autism’ and ‘Asperger’, and to identify news and trending topics related to ASD that took place during the weeks with the highest number of Google searches for these terms.”	Autism, Asperger, Campaign, News headline impact	
“We sought to identify correlations in Google searches (Google LLC, Mountain View, California) for rosacea and comorbid conditions to assess whether the public is seeking information regarding these trends.”	Rosacea, Comorbid conditions, Information seeking behavior, Public interest	Online Behavior and Mental Health
“Our aim is to investigate Internet search interest across construction with a focus on mental, stress and suicide, and determine whether there is consistent interest across these search terms.”	Internet search, Mental health, Stress, Suicide	
“The goal of this study was to examine whether statewide mask mandates and political party affiliations yielded differential changes in mental health symptoms across the United States by leveraging state‐specific Internet search query data.”	Legislation, Mental health, Public interest	Public Health Policies and Government Responses
“This infodemiological study utilized Relative Search Volumes (RSV) from Google Trends. It determined changes in public interest in mental health after the implementation of the mental health laws of Malaysia, the Philippines, Singapore, and Thailand using search volumes from 2004 to 2021.”		
“To analyze and understand the Philippine government's response to the COVID‐19 pandemic, characterized by policy experimentation and incremental adaptation.”	Government response, COVID‐19	
“It is fair to assume that the issued NPIs have heavily affected social life and psychological functioning. We therefore aimed to examine possible effects of this lockdown in conjunction with daily new infections and the state of the national economy on people's interests, motives, and other psychological states.”	COVID‐19, Psychological effects, Social life, Economic impact	Psychological and Social Impacts
“This study aims to identify global trends in social prescribing from 2018. To this end, we intend to collect and analyze words related to social prescribing worldwide and evaluate various trends of related words by classifying the core areas of social prescribing.”	Social prescribing	
“In a view to studying impacts on student's fraternity, this article aims at addressing alternative ways of educating‐more specifically, online education‐through the analysis of Google Trends for the past year.”	Student fraternity	
“Aimed to address the phenomenon of celebrities’ impact on public apprehension, revise the syndrome for the medical community, and emphasize taking advantage of such involvement of celebrities for improving the spread of highly important medical information for the public. © 2023 by the authors.”	Public awareness, Public interest, Celebrity impact	
“We hypothesized that social media challenges for particular substances would temporally correspond with increased ingestions of these substances.”	Social media, Substance	
“This study analyzed public concern regarding gambling as a health topic using aggregated Google searches. Using an infodemiological design, a search query using the keyword ‘Gambling (Topic)’ was done in Google Trends.”	Gambling	
“The present study explored the potential for Internet search data to serve as indicators of subjective well‐being (SWB) and predictors of health at the state and metro area levels.”	Well‐being	

### Content analysis

#### Thematic analysis of the research objectives

The thematic analysis of research objectives utilizing Google Trends data on mental health has revealed a diverse array of six themes.

##### Economic and social impact on mental health (12 codes)

This theme encompasses research examining the effects of economic factors, such as unemployment, financial instability, and stock market fluctuations on mental health. Studies also explore mood changes, cultural variations, and discrimination. This theme highlights the significant interplay between economic conditions and mental well‐being. Overall, 12 studies fall under this theme.

##### Mental health during public health emergencies (9 codes)

This was the most extensive theme, comprising 54 studies focusing on mental health during public health crises, primarily the COVID‐19 pandemic. This theme covers psychological effects, including insomnia, suicide, psychological impacts, lockdowns, and overall well‐being.

##### Online behavior and mental health (5 codes)

This theme with 11 studies investigates the relationship between Internet search behavior, public interest, stress, and information‐seeking behavior. This theme highlights the growing relevance of digital behavior in understanding mental health trends and public interest during various crises.

##### Specific mental health conditions and treatment (20 codes)

Encompassing 51 studies, this theme focuses on specific mental health conditions, such as anxiety, depression, suicide, substance use, ADHD, eating disorders, schizophrenia, and bipolar disorder. It also covers the treatment aspects, including the use of psychotropic medications, antidepressants, anxiolytics, and psychiatric hospitalization.

##### Public health policies and government responses (4 codes)

This theme includes nine studies examining the role of legislation, public health policies, and government responses in addressing mental health issues. The research within this theme often explores the adoption of specific health policies, such as the use of hydroxychloroquine (HCQ), and its implications for mental health.

##### Psychological and social impact (9 codes)

Covering 23 studies, this theme delves into the psychological and social impacts of various factors, including social networks, social media, gun violence, politics, celebrity influence, emotional well‐being, and overall happiness. The studies highlight the broad spectrum of social determinants influencing mental health (Table 1).

### Specific health conditions

We calculated the number of studies on specific health conditions. The conditions most frequently studied include mental health concerns with COVID‐19 (61 studies), anxiety,^12^ depression,^13^ and suicide (*n* = 33). Other conditions, such as substance use (*n* = 5), bipolar disorder (*n* = 2), schizophrenia (*n* = 2), autism (*n* = 1), ADHD (*n* = 1), eating disorders (*n* = 1), and Asperger syndrome (*n* = 1), are also covered but to a lesser extent.

## DISCUSSION

This study aimed to analyze the research landscape of Google‐Trends‐based mental health studies through bibliometric and content analyses. The bibliometric analysis revealed upward trends (especially during and after the pandemic) in publication volume, collaboration networks, and thematic focuses among journals, authors, countries, and organizations. The content analysis identified six major themes reflecting the diverse objectives of these studies, ranging from the economic and social impacts on mental health to specific mental health conditions and their treatment. Key mental health conditions frequently studied included COVID‐19, anxiety, depression, and suicide, indicating a strong research focus on these areas.

### Geographical and temporal variations in themes

The bibliometric analysis identified three major research clusters based on geographic and thematic variations in Google‐Trends‐based mental health studies. North American and European research largely concentrated on suicide prevention and public health surveillance, while Asian and Middle Eastern studies focused on mental health stigma and treatment‐seeking behaviors. Latin America and Africa had relatively fewer studies, with an emphasis on substance use disorders and mental health accessibility. Temporal trends indicated an evolution from early studies on seasonal variations in depression searches to recent explorations of COVID‐19‐related psychological distress and digital mental health trends.

### Implications of the bibliometric indices

The bibliometric analysis provided valuable insights into the development and collaboration patterns within the field of Google‐Trends‐based mental health research. Bibliographic coupling analysis highlighted the interconnectedness of research communities, with prominent clusters centered around digital health, public health, and environmental factors affecting mental health. This suggests that journals like the *Journal of Medical Internet Research* play a crucial role in disseminating research on digital health and public health surveillance.^1^ The concept of bibliographic coupling has also been instrumental in identifying research trends in other fields, such as climate change and infectious diseases, indicating its broad applicability.^14^ Keyword concurrence analysis underscored the centrality of mental health issues related to the COVID‐19 pandemic, with keywords like “mental health,” “depression,” “anxiety,” and “social media” frequently co‐occurring. This highlights the significant psychological impacts of the pandemic and the role of digital behavior in mental health research.^12^, ^15^ Similar patterns have been observed in other domains, where keyword analysis revealed critical themes and research priorities, such as in environmental health and social sciences.^16^ Author co‐citation analysis revealed two main clusters of frequently cited authors, indicating limited collaborative research communities focused on mental health themes. Co‐citation analysis is a well‐established method in bibliometrics, often used to identify influential researchers and seminal works within a field. For example, in studies of global health and digital epidemiology, co‐citation analysis has similarly identified key figures and foundational publications that shape the research landscape.^17^, ^18^ Author coauthorship analysis further emphasized minimal collaborations, with closely knit networks of authors contributing to the field. This type of analysis has been crucial in understanding the dynamics of scientific collaboration and the development of research networks in various disciplines, including biomedical sciences and public health.^19^ The strong collaborative ties identified in this study highlight the importance of interdisciplinary efforts in advancing mental health research. Coauthor country analysis showed a few international collaborations, particularly involving the United States, the United Kingdom, and Italy, reflecting limited global interest and collaborative efforts in this research area.^13^ International collaboration is a common feature in high‐impact research areas, facilitating the exchange of knowledge and resources across borders. Although significant global collaborations have been documented in fields such as infectious disease research and climate science, this is not visible for mental health research, underscoring the value of international partnerships in addressing complex global challenges.^20^ The application of Google Trends for mental health research is relatively novel and the analytics are still evolving, which might explain limited interest and collaborations around this line of work. There is a relative dearth of funding in Google‐Trends‐based mental health research. Interestingly, there was a single study funded by Google. Open access to the data reduces the need for funding, but the novelty of analytics and the exploratory nature of the research might also deter established funders.

### Implications of the content analyses

The content analysis provided a deeper understanding of the specific objectives driving Google‐Trends‐based mental health research. The six identified themes—economic and social impacts, mental health during public health emergencies, online behavior and mental health, specific mental health conditions and treatment, public health policies and government responses, and psychological and social impacts—underscore the multifaceted nature of this research.^1^, ^20^

Studies exploring the economic and social impacts on mental health highlight the critical interplay between financial instability and mental well‐being, suggesting the need for targeted interventions during economic downturns.^14^ Research on mental health during public health emergencies emphasizes the profound psychological effects of crises like the COVID‐19 pandemic, indicating the importance of timely mental health support and public health preparedness.^15^ The focus on online behavior and mental health reflects the growing relevance of digital behavior in understanding mental health trends and public interest during various crises.^17^, ^19^

Studies on specific mental health conditions and treatment provide insights into the prevalence and treatment strategies for conditions like anxiety, depression, and suicide, informing clinical practice and public health policies.^12^ The examination of public health policies and government responses highlights the role of legislative and policy measures in addressing mental health issues, emphasizing the need for evidence‐based policy‐making.^13^ Finally, research on the psychological and social impacts of various factors underscores the broad spectrum of determinants influencing mental health, suggesting the need for holistic approaches in mental health interventions.^16^, ^18^

## LIMITATIONS

This study has several limitations. The reliance on a single database search results may introduce selection bias, potentially overlooking relevant studies not indexed in the selected databases. Our search strategy was not designed to capture research on substance misuse. The exclusion of non‐English publications may also result in a language bias, limiting the generalizability of the findings to a global context. Additionally, there are inherent issues with the Google Trends data; while innovative, these may not fully capture the complexity of mental health issues, as search behavior may be influenced by various factors unrelated to actual mental health conditions. The content analysis was based on the study objectives in the abstract; hence, the content and quality of reporting might affect the robustness of the qualitative analysis. Although connected maps provide a broad overview of the entire research landscape, showing how different elements are related, the interconnected nature can sometimes blur the boundaries between different clusters, making it harder to distinguish distinct research areas. Applying minimum thresholds may result in excluding nodes that, while not highly cited or frequently occurring, are still relevant to the research context. Minimum number specifications favor well‐established authors, institutions, and topics, potentially biasing the analysis towards existing power structures and overlooking innovative or niche research areas.

Google Trends has several limitations as a research tool. First, search behavior does not necessarily reflect clinical diagnoses, and individuals with conditions such as schizophrenia, bipolar disorder, or personality disorders may be underrepresented in search data. Second, search trends are influenced by media coverage, seasonal changes, and stigma, leading to potential misinterpretation. Additionally, ambiguity in keyword selection and variations in Google's search algorithms may introduce inconsistencies over time. Google trends could be used beyond the current research themes.^21^

## RECOMMENDATIONS

Future Google‐Trends‐based research on mental health should focus on several key areas. First, researchers should address underrepresented topics, such as mental health in specific age groups, like adolescents or the elderly, as well as underexplored mental health conditions, like eating disorders, schizophrenia, or autism. Expanding the scope of research to include these areas would provide a more comprehensive understanding of public interest and concerns. Second, integrating Google Trends with demographic data can offer insights into mental health trends among different age groups, genders, or ethnic communities. This approach would help tailor public health interventions to specific populations. Google Trends itself does not directly provide data segmented by age groups, genders, or ethnic communities; however, the addition of geographic proxies, social media surveys, and cross‐referencing (e.g., census reports) could help explore different demographics. Third, researchers should consider the role of economic and social factors in mental health, particularly during times of financial instability or public health emergencies, as these themes emerged prominently in the content analysis. Additionally, interdisciplinary collaboration should be strengthened, as the bibliometric analysis highlighted limited collaboration among researchers and institutions. By fostering partnerships across disciplines and countries, researchers can leverage diverse expertise and resources, leading to more robust and impactful studies. Mechanisms to encourage global collaboration include establishing multinational research consortia, developing open‐access datasets, and promoting interdisciplinary research teams. Increased collaboration between high‐income and low‐income regions can enhance the generalizability of Google‐Trends‐based mental health research and contribute to more equitable global mental health policies.^22^ Finally, securing funding from diverse sources, including government agencies, foundations, and private companies, is crucial to support the expansion of Google‐Trends‐based mental health research, especially given the relatively low levels of funding currently observed.

## SUMMARY AND CONCLUSION

The bibliometric analyses emphasize the critical role of digital health research, international collaborations, and key institutions in advancing this field. The content analysis reveals diverse research objectives, underscoring the importance of economic, social, and psychological factors in mental health research. Despite its limitations, this study contributes valuable insights into the current state of Google‐Trends‐based mental health research, providing a foundation for future studies to build upon.

## AUTHOR CONTRIBUTIONS

The authors contributed to this study as follows: Abhishek Ghosh and Sonu Goel conceptualized and designed the study, playing a central role in guiding its direction. Abhishek Ghosh, along with Blessy B. George and Pragyapti Malav, conducted the data curation, formal analysis, and investigation, ensuring that the research was thorough and comprehensive. Blessy B. George and Pragyapti Malav were additionally supported by the Indian Council of Medical Research as Project Scientists, providing essential funding for the study. For the methodological framework, Abhishek Ghosh, Blessy B. George, and Pragyapti Malav collaborated closely to ensure a solid and reliable foundation for the research. Shinjini Choudhury and Sonu Goel provided critical supervision and oversight throughout the project, helping guide the study from conception to completion. Both Shinjini Choudhury and Sonu Goel were involved in the project administration, ensuring that the research adhered to its objectives and timeline. The manuscript preparation was a joint effort by all authors. Abhishek Ghosh, Blessy B. George, Pragyapti Malav, Shinjini Choudhury, and Sonu Goel contributed to drafting the original manuscript, with Shinjini Choudhury and Sonu Goel performing the final review and editing. Their collective efforts ensured a high standard of academic rigor and integrity in the final document.

## CONFLICT OF INTEREST STATEMENT

The authors declare no conflicts of interest.

## ETHICS APPROVAL STATEMENT

As this study involved the analysis of publicly available data and published literature, it did not require formal ethical approval.

## PATIENT CONSENT STATEMENT

N/A.

## CLINICAL TRIAL REGISTRATION

N/A.

## Supporting information

Appendices 1.

## Data Availability

Data sharing does not apply to this study as it is based on secondary literature.
