# Supplementary material for: Mapping the landscape of mental health research through Google Trends: Bibliometric and thematic insights
Source: PCN Rep. 2025 Apr 27;4(2):e70101. doi: 10.1002/pcn5.70101 (PMC12034257; doi:10.1002/pcn5.70101)
Supplement: Supplementary file 1 — Appendices 1. [file PCN5-4-e70101-s001.docx]

**Appendices/ Supplementary files**

**Supplementary figures**

**Supplementary Figure 1 Publication Trends**

**Supplementary Figure 2 Author-Coauthor Collaboration**


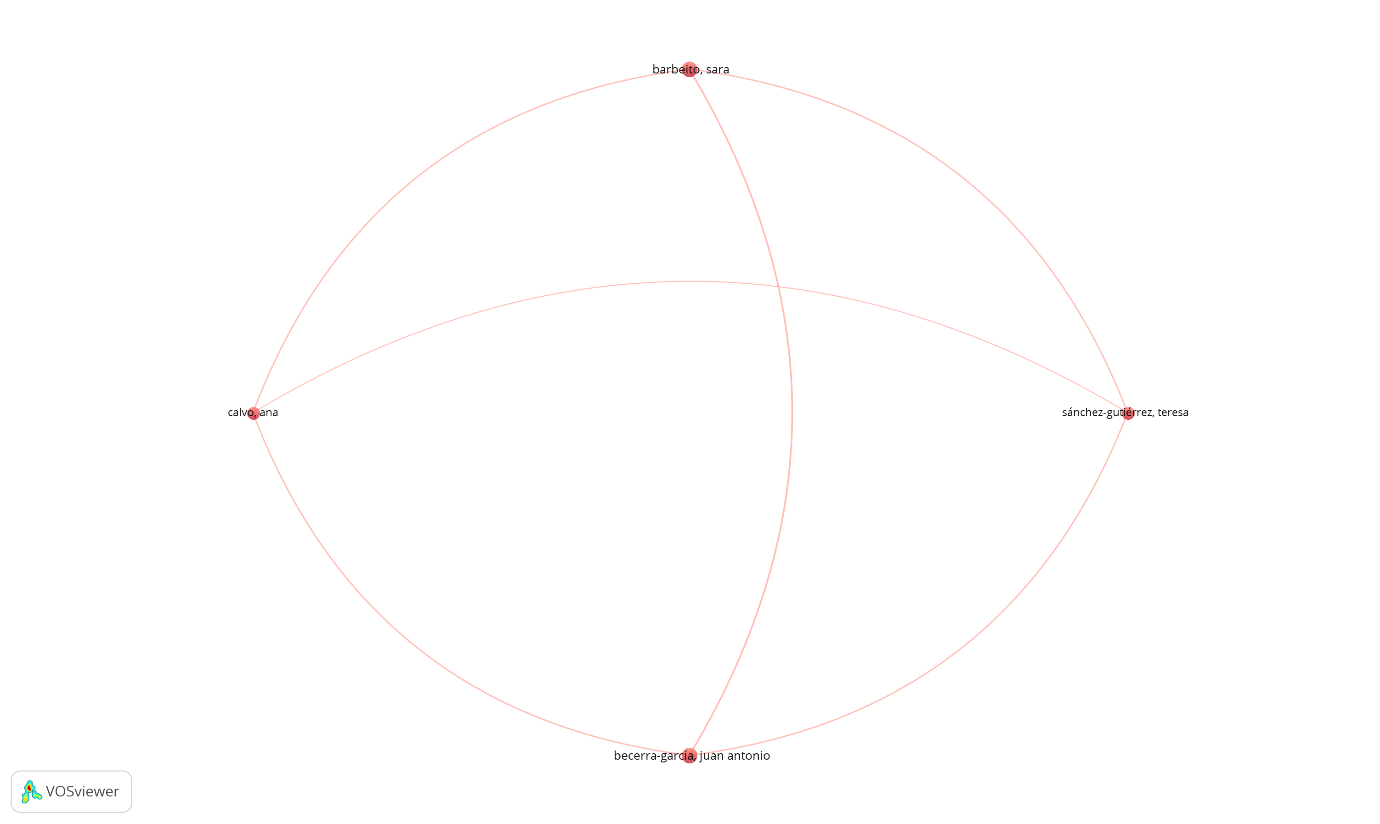


This figure illustrates a co-authorship network among four authors: Sara Barbalito, Teresa Sánchez-Gutiérrez, Juan Antonio Becerra-García, and Ana Calvo. The connecting lines indicate strong collaborative relationships, highlighting a closely-knit research group within the mental health field.

**Supplementary Figure 3 Author-Citation Analysis**


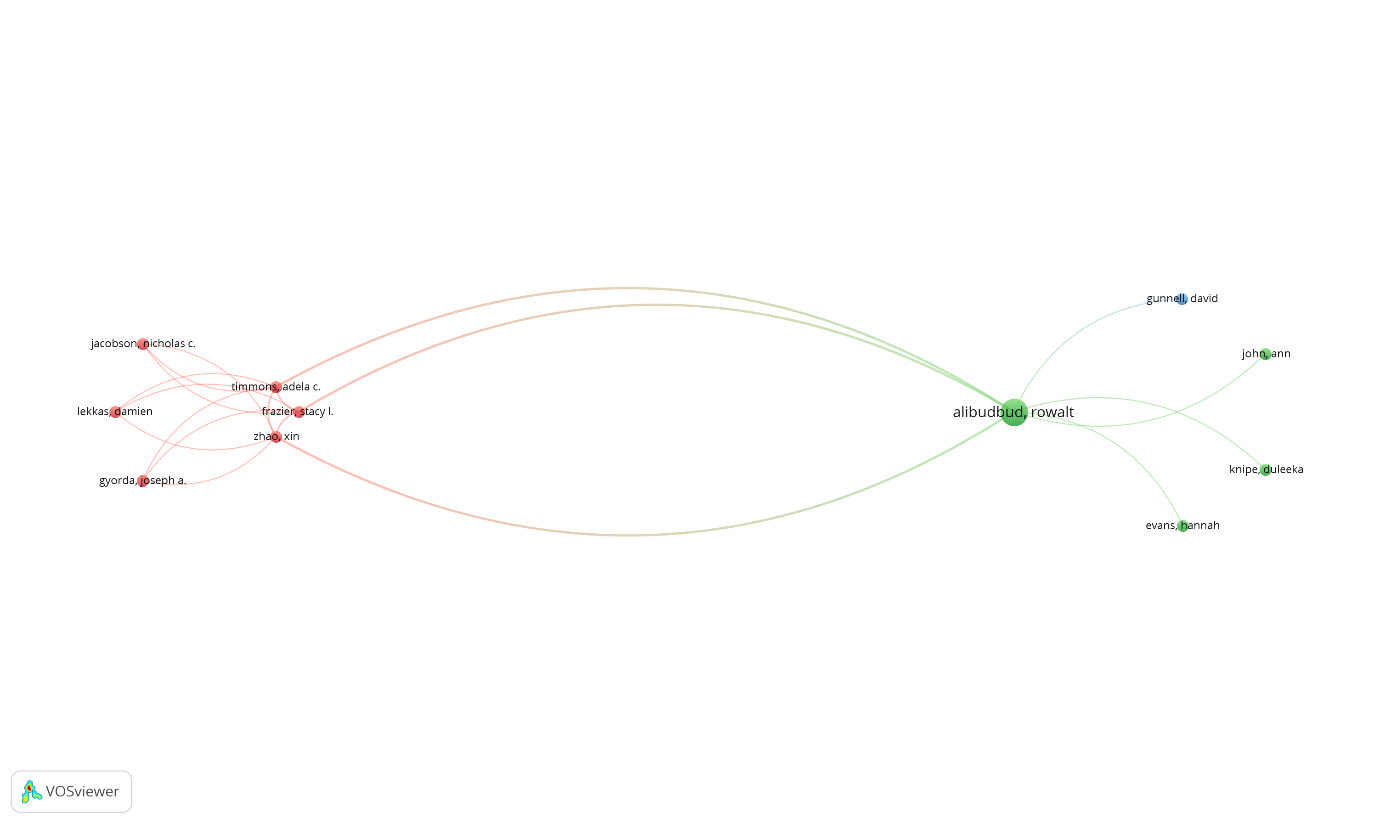


The network visualization illustrates author co-citation relationships in Google Trends-based mental health research. Nodes represent authors, with size indicating citation frequency. Edges signify co-citation links, where thicker lines denote stronger co-citation connections. The figure reveals two main clusters: the red cluster, including Nicholas C. Jacobson and Adela C. Timmons, suggesting frequent co-citations and collaborative research focus, and the green cluster centered around Rowalt Alibudbud, indicating strong internal co-citation links and significant influence in the field.

**Supplementary Figure 4 Co-Author Organization Analysis**


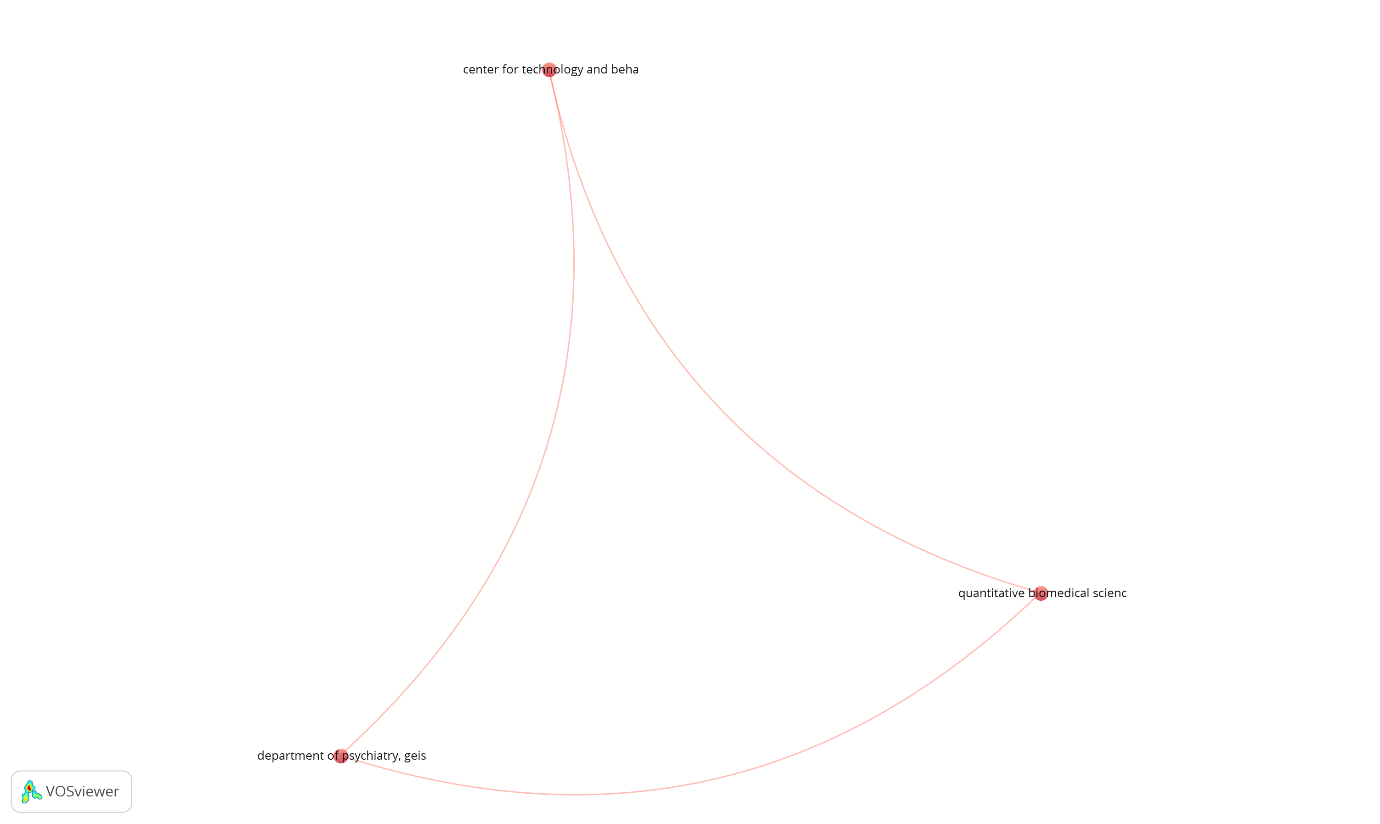


This figure shows the collaboration among organizations in mental health research using Google Trends data. Each node represents an organization, with size indicating publication frequency. Thicker edges indicate stronger collaborations. Highlighted organizations include the Center for Technology and Behavior, Department of Psychiatry at Geis, and Quantitative Biomedical Sciences, indicating significant partnerships among them.

**Supplementary: Funding Support**

**Government Agencies - 45**

- National Institute for Health Research
- National Institute on Drug Abuse
- National Institutes of Health
- European Regional Development Fund
- National Natural Science Foundation of China
- Australian Government
- Australian Research Council
- Biotechnology and Biological Sciences Research Council
- Department of Science and Technology, Government of Kerala
- Economic and Social Research Council
- Engineering and Physical Sciences Research Council
- European Research Council
- Grantová Agentura České Republiky
- Horizon 2020 Framework Programme
- Ministerio de Asuntos Económicos y Transformación Digital, Gobierno de España
- Ministerio de Economía y Competitividad
- Ministerio de Educación, Cultura y Deporte
- Ministero della Salute
- Ministero dell’Istruzione, dell’Università e della Ricerca
- Ministry of Education
- Ministry of Education of the People's Republic of China
- Ministry of Science and Technology, Taiwan
- NIHR School for Primary Care Research
- Narodowe Centrum Nauki
- National Center for Advancing Translational Sciences
- National Health and Medical Research Council
- National Human Genome Research Institute
- National Institute of Allergy and Infectious Diseases
- National Institute of General Medical Sciences
- National Institute of Mental Health
- National Institute on Minority Health and Health Disparities
- National Research Foundation
- National Research Foundation of Korea
- Research Councils UK
- Research Grants Council, University Grants Committee
- UK Research and Innovation
- Institut National de la Santé et de la Recherche Médicale

**Universities and Research Institutions - 23**

- Cardiff University
- City University of Hong Kong
- Faculty of Medicine and Dentistry, University of Alberta
- Florida International University
- Hallym University
- Herbert Wertheim College of Medicine, Florida International University
- Heriot-Watt University
- INTI International University and Colleges
- Keio University
- Laurea University of Applied Sciences
- Universidad Internacional de La Rioja
- University Graduate School, Florida International University
- University Hospitals Bristol NHS Foundation Trust
- University of Alberta
- University of Bristol
- University of Hong Kong
- University of Texas MD Anderson Cancer Center
- University of Toronto
- University of the Philippines
- Università degli Studi di Padova
- Université Paris-Est Créteil Val-de-Marne
- Office of the President, University of California
- Center for Coastal Oceans Research, Florida International University

**Foundations and Trusts - 12**

- Wellcome Trust
- Academy of Finland
- Agencia Nacional de Investigación y Desarrollo
- Fundación Alicia Koplowitz
- Koneen Säätiö
- Lietuvos Mokslo Taryba
- Medical Research Council
- Nuffield Foundation
- Robert Wood Johnson Foundation
- Science Foundation Ireland

**Private Companies and Organizations - 7**

- Assistance Publique - Hôpitaux de Paris
- FURTHERMORE grants in publishing
- Google
- Idorsia Pharmaceuticals
- Oklahoma Center for the Advancement of Science and Technology
- Prix Inspiration Arctique
- Sanofi
